# Insight into microRNAs’ involvement in hematopoiesis: current standing point of findings

**DOI:** 10.1186/s13287-023-03504-3

**Published:** 2023-10-04

**Authors:** Seyed Mahdi Nassiri, Neda Ahmadi Afshar, Parsa Almasi

**Affiliations:** Department of Clinical Pathology, Faculty of Veterinary Medicine, University of Tehran, Qarib St., Azadi Ave, Tehran, Iran

**Keywords:** Differentiation, Myelopoiesis, Hematopoiesis, miRNA

## Abstract

Hematopoiesis is a complex process in which hematopoietic stem cells are differentiated into all mature blood cells (red blood cells, white blood cells, and platelets). Different microRNAs (miRNAs) involve in several steps of this process. Indeed, miRNAs are small single-stranded non-coding RNA molecules, which control gene expression by translational inhibition and mRNA destabilization. Previous studies have revealed that increased or decreased expression of some of these miRNAs by targeting several proto-oncogenes could inhibit or stimulate the myeloid and erythroid lineage commitment, proliferation, and differentiation. During the last decades, the development of molecular and bioinformatics techniques has led to a comprehensive understanding of the role of various miRNAs in hematopoiesis. The critical roles of miRNAs in cell processes such as the cell cycle, apoptosis, and differentiation have been confirmed as well. However, the main contribution of some miRNAs is still unclear. Therefore, it seems undeniable that future studies are required to focus on miRNA activities during various hematopoietic stages and hematological malignancy.

## Introduction

**Fig. 1:**
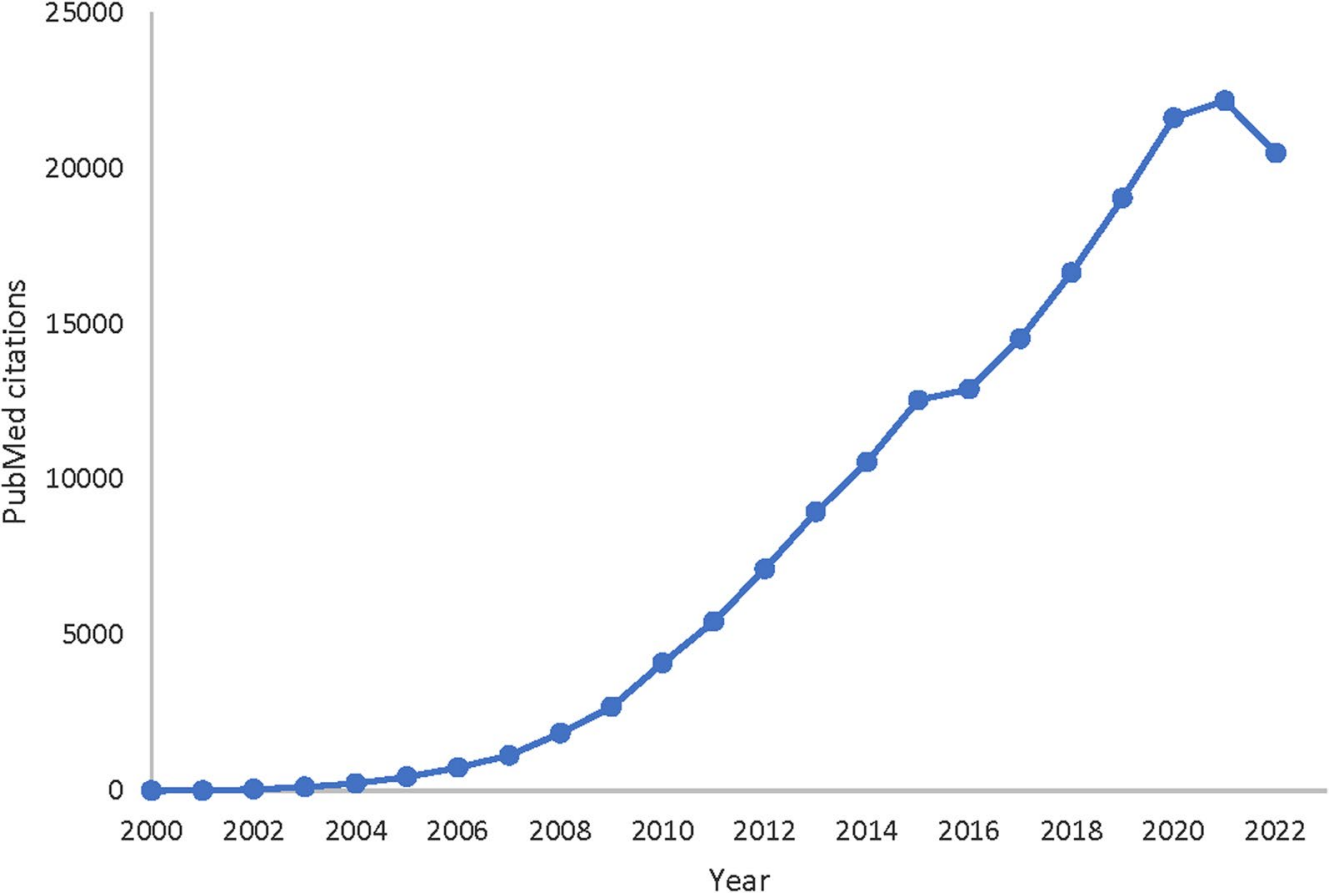
Number of published papers containing “microRNA” or “miRNA” keywords in the PubMed database during 2000–2022

The formation, development, and differentiation of blood cells occur under a regular and complex process known as hematopoiesis, which can be regulated by various mechanisms, including miRNAs [1]. miRNAs were discovered in 1993 as a group of small single-stranded RNA molecules, binding to complementary sequences in mRNA molecules. Longer RNA precursors are miRNA generation sources, produced by cellular enzymes. These molecules are a type of single-stranded RNA composed of about 22 nucleotides, forming a complex with at least one protein. The complex binds to any mRNA molecule with at least seven/eight complementary nucleotide sequences. The protein-miRNA complex degrades the target mRNA; however, in some rare cases, it blocks the translation process. It has been revealed that approximately 1,500 encoding genes are being controlled by miRNAs in the human genome. Biologists suggest that miRNAs may regulate the expression of at least half of the human genes [2]. The evaluation of PubMed citations during the last decades demonstrates the importance of miRNAs with an uprising trend of published papers in this regard in recent years (Fig. 1).

During the last decades, various studies have attempted to uncover different roles of miRNAs in the hematopoiesis process [1, 3–8]. For instance, Kim et al. (2019) reported that miRNAs play a key role as the inhibitors of hematopoietic transcription factors, while their involvement in red cell physiology has been elucidated as well [9]. Moreover, some researchers have discussed miRNA participation in several possible erythrocyte-derived pathways [10].

Despite previous evidence, various functional aspects and underlying mechanisms of miRNAs in hematopoiesis remain unknown [10]. For instance, it has been found that miR-18 and some other miRNAs have distinct functions in regulating different target genes, which should be identified more accurately [11]. Similarly, contradictory findings have been reported regarding miR-24 function in the hematopoiesis process [8]. Further, the key role of miRNAs in inhibiting blood cell apoptosis is still unclear [12].

## miRNA regulation in hematopoietic stem cells (HSCs) (Table 1)

**Table 1:** miRNAs expressed in HSCs with details

miRNA	Functions	Target	References
miR-146a	Blocking differentiation	Suppressing IRAK1 and TRAF6	Bissels et al. [21]
miR-10a	Blocking differentiation	ND	
miR-29a	Regulating extracellular matrix	ND	
	Repressing apoptosis	Blocking BAK1	
miR-29b	Regulating extracellular matrix	ND	
miR-23	Inhibiting B-cell differentiation	Blocking B-cell receptor	
miR-24	Inhibiting B-cell differentiation	Blocking B-cell receptor	
	Inhibiting apoptosis	Suppressing caspase 9 and ALK4	
miR-125a/b	Inhibiting B-cell differentiation	Blocking B-cell receptor	
	Inhibiting apoptosis	Blocking BAK1 and p53	
	Remodeling cytoskeleton	ERBB2 and ERBB3	
miR-142-5p	Blocking differentiation	Suppressing UPS	Bissels et al. [21]
miR-142-3p			
miR-191			
miR-484			
miR-425			
miR-17	Blocking differentiation at early	ND	Georgantas et al. [23]
miR-24	progenitor stage		
miR-128			
miR-146			
miR-155			
miR-181			
miR-16	Blocking differentiation at later	ND	Georgantas et al. [23]
miR-103	progenitor stage		
miR-107			
miR-221 miR-222	Protecting HSC quiescence and pluripotency	Reducing the expression of IEG	Jani et al. [24]
miR-223	Blocking differentiation at terminal progenitor stage	ND	Georgantas et al. [23]
miR-105	MK differentiation in human embryonic stem cell and adult CD34 + cell	Suppressing c-Myb	Kamat et al. [25]
miR-193b	Limiting self-renewal and proliferation	Regulating MAPK/ERK	Issa et al. [26]
miR-Let-7	Inhibiting self renewal	Blocking HMGA2	Copley et al. [27]
miR-99a/100 ~ 125b	Promoting self renewal	Blocking TGF-β	Emmrich et al. [28]
	Increasing proliferation	Upregulating Wnt signaling	
miR-22	Promoting self renewal	Suppressing TET2	Song et al. [29]
	Blocking differentiation		
miR-126	Blocking differentiation	Reducing signal transduction in	Lechman et al. [30]
	Impairing cell cycle	PI3K/AKT/GSK3β pathway	
miR-139-3p	Blocking differentiation	Suppressing EIF4G2	Emmrich et al. [31]
	Decreasing proliferation	Suppressing HuR	Alemdehy et al. [32]
miR-199a-3p	Increasing proliferation	Prdx6, RUNX1 and Suz12	Alemdehy et al. [32]
miR-212/132 (miR-19)	Accelerating cell cycle	Regulating the expression of FOXO3	Mehta et al. [33]
	Destruction of HSCs		
	Maintaining the balance of hematopoiesis		
miR-29a/b-1	Promoting self renewal	Suppressing DNMT3A	Hu et al. [34]
	Inhibiting apoptosis		
	Decreasing cell cycle rate		
miR-33	Dysregulation of self-renewal	Blocking p53	Herrera-Merchan et al. [36]
miR-125b	Expansion of early HSCs	Suppressing proapoptotic factors Bmf and KLF13	Ooi et al. [38]

PBSC: Peripheral blood stem cell; BM: Bone marrow; ND: Not determined

HSCs are undifferentiated immortal cells, producing different classes of blood cells in addition to self-renewal [13–15].

CD133, also called Prominin-1, is expressed in HSC and hematopoietic progenitor cell. These cells were derived from the human fetal liver, bone marrow (BM), peripheral blood, and cytokine-mobilized peripheral blood progenitor cells [16–18]. Additionally, CD133 + cells in the quiescent phase are phenotypically similar to HSCs, with a high self-renewal ability [19]. It was also revealed that CD34 + cells can be generated in vitro from CD133 + CD34 + cells, indicating that CD133 + cells might be the probable ancestor cells of CD34 + cells [20].

The miR-10a, miR-125b, miR-146a, miR-125a-5p, miR-551b, miR-99a, miR-29a, miR-146b-5p, miR-29b, miR-29c, miR-23a, miR-24, and miR-23b were reported as higher expressed miRNAs in marrow-derived CD133 + HSCs when compared to CD34 + CD133– HSCs. However, miR-142-5p, miR-191, miR-142-3p, miR-484, and miR-425 in the CD133 + cells were demonstrated to be lower expressed in comparison to CD34 + CD133– cells [21].

### miRNAs involved in HSC differentiation

The functional analysis of the above-mentioned miRNAs expressed in CD133 + HSCs has shown their key role in hematopoietic or lymphoid organ development. Overexpression of miR-146a and miR-10a was found to prevent megakaryopoiesis in human-derived CD133 + HSCs. Also, miR-146a inhibited cell differentiation by blocking IRAK1 and TRAF6 transcription factors. MiR-29a and miR-29b participated in the cytoskeleton organization by regulating the extracellular matrix. In addition, miR-29a was shown to negatively regulate BCL2 antagonist/killer 1 (BAK1), exerting an anti-apoptotic role in cells. Moreover, miR-23, miR-24, and miR-125a/b inhibited B-cell differentiation through preventing B-cell receptor signaling. Additionally, miR-24 was capable of prohibiting caspase 9 (a proapoptotic factor) and activin-like kinase 4 (ALK4), then repressing apoptosis and erythroid differentiation. MiR-125a/b could also have an anti-apoptotic role by targeting p53 and BAK1. In addition, miR-125a/b participated in cytoskeleton remodelling through targeting ERBB2 and ERBB3 [21].

The key biological role of miR-142-5p, miR-191, miR-142-3p, miR-484, and miR-425 in the CD34 + CD133– HSCs was found to target ubiquitin–proteasome system (UPS) and block cell differentiation. The UPS is known as an important regulator of protein stability and activity with a pivotal role in HSC maintenance and differentiation, where reduced UPS led to HSC differentiation and leukemogenesis [21]. Similarly, Moran-Crusion et al. (2012) concluded that mutations or loss of UPS could lead to leukemia [22].

Accordingly, a study conducted on human BM and peripheral blood-derived CD34 + hematopoietic stem-progenitor cells (HSPCs) reported a number of overexpressed miRNAs (listed in Table 1). However, no specific miRNAs were detected to contribute to multipotential progenitor (MPP) development during the HSC. It was represented that miR-17, −24, −146, −155, −128, and −181 inhibited (maintained) early hematopoietic cells at an early stem-progenitor stage and blocked their differentiation into mature cells. Although miR-16, −103, and −107 may also block later progenitor cells’ differentiation, it has been suggested that miR-221, −222, and −223 most likely regulate the terminal phases of hematopoietic differentiation. Additionally, miR-155 was demonstrated as a potent inhibitor of HSPC differentiation [23]. Jani et. al showed that miR-221/222 directly targets FOS and indirectly JUN as well as several other immediate early genes such as immediate early response 2 (IER2), lF6, JUNb, Kruppel-like factor 6 (KLF6), nuclear receptor 4A1 (NR4A1) and zinc finger protein 36 (ZFP36) in a mice model. They revealed that the stress and absence of miR-221/222 expression drive HSCs towards MPP by increasing Fos/AP-1/IEG expression, leading to cell cycle progression and granulopoiesis. Also, this study determined that in the absence of miR-221/222, HSC retained the capacity of homing and dormancy in the bone marrow but lost its multipotency. Overall, it was shown that the expression of miR-221/222 in HSC and MPP protects their quiescence and pluripotency by reducing the expression of IEG and myelo/granulopoiesis enhancing target genes [24]. Moreover, miR-105 participates in human embryonic stem cells and adult CD34 + cells. miR-105 enhanced megakaryopoiesis in both progenitors by reducing the hematopoietic transcription factor c-Myb [25].

One of the essential features of miRNAs is their anti-tumor properties that can be used to treat malignancies. miR-193b, an endogenous tumor suppressor, regulates several members of the RAS-RAF-MEK-ERK (MAPK/ERK) cascade, thereby regulating proliferation and cell cycle progression. MAPK/ERK is activated during differentiation but remained suppressed in HSCs. The (THPO)-MPLSTAT5 signalling cascade in HSCs increases the expression of miR-193b and limits self-renewal and proliferation, thereby preventing the exhaustion of HSCs [26]. It was shown in a mice model that the clinical use of miR-193b-encapsulated lipid nanoparticles can improve the efficacy and tolerance of current chemotherapy approaches in hematopoietic neoplasia related to mutations in miR-193b factors [26].

### miRNAs involved in HSC self-renewal and cell cycle

Let-7 played a role in HSC self-renewal through the Lin28b-Let-7-HMGA2 axis. In mouse embryonic HSCs, the expression of Lin28B and high mobility group A2 (HMGA2) is higher than in adult HSCs, whereas the expression of some miRNAs of the Let-7 family is the opposite. The high expression of Lin28B and HMGA2 in HSCs resulted in increased self-renewal of these cells. However, Let-7 inhibits this process by targeting HMGA2, which is blocked by Lin28B. [27].

In a study on human cord blood HSCs/HSPCs and transgenic mice, Emmrich and colleagues discovered a common regulatory function of miR-99a/100, Let-7 and miR-125b homologs. This study determined that miR-99a/100 ~ 125b tricistrons were produced from a primary transcript activated by Homeobox A10 (HOXA10). Coordinated activity of all three tricistronic miRNAs led to the expansion of HSCs and MPs by jointly blocking the transforming growth factor beta (TGF-β) pathway and promoting Wnt signaling. TGF-β and Wnt pathways were reported as principal regulatory signaling cascades. TGF-β led HSCs to enter in the quiescence and differentiation phases, whereas Wnt pathways led them to self-renewal and proliferation. In general, the HOXA10- miR-99a/100 ~ 125b- TGF-β/Wnt axis took part in the development and differentiation of HSCs (with a preference for megakaryopoiesis) and leukemogenesis [28].

Meanwhile, it was found that HSCs of transgenic mice with miR-22 expression showed an increased self-renewal with impaired differentiation. Contrariwise, inhibition of miR-22 blocked proliferation in murine and human leukemia cells [29]. This study also showed that one of the critical targets of miR-22 in this context is tet methylcytosine dioxygenase 2 (TET2) as the ectopic expression of TET2 suppressed the phenotypes induced by miR-22. Downregulation of TET2 protein is also associated with poor clinical outcomes with overexpression of miR-22 in MDS patients. Therefore, miR-22 was introduced as a strong proto-oncogene common in the miR-22/TET2 regulatory pathway in hematopoietic malignancies [29].

One of the important miRNAs associated with interfering HSCs cell cycle progression in vitro and in vivo is miR-126. In a study, the knockdown of miR-126 using lentiviral sponges in mouse and human HSCs led to an uninterrupted proliferation of HSCs. Also, the mentioned study determined that enforced expression of miR-126 resulted in the differentiation inhibition of HCSs in hematopoiesis. Indeed, miR-126 had a key role in controlling the function of HSCs and accomplished this function by targeting the PI3K/AKT/GSK3β pathway. In this way, it reduced signal transduction in response to external signals and set a threshold for HSC activation. Therefore, its ultimate goal is to control the size of the HSC pool [30].

Typically, miR-139-3p is expressed in terminally differentiated neutrophils and macrophages. Ectopic expression of miR-139-3p in normal CD34 + hematopoietic stem and progenitor cells was found to result in disruption of myelomonocyte differentiation and suppression of proliferation in a xenograft mouse model of human cell lines representing major AML subgroups. miR-139-3p reduced the overall protein synthesis by suppressing the translation initiation factor EIF4G2, thereby exerted its biological effects [31]. Also, the other study by Alemdehy et al. 2015 displayed that ectopic expression of miR-139-3p repressed the proliferation of myeloid progenitors by targeting HuR. As a matter of fact, miR-139-3p, as a global tumor suppressor-miR in AML, can be used as a new way to treat AML. Contrarily, miR-199a-3p was found to target Prdx6, RUNX1 and Suz12, leading to the proliferation of mice myeloid progenitors [32]. Overall, the enforced expression of miR-199a-3p induced AML in a pre-leukemic mouse model, presenting it as an onco-miRNA [31, 32].

Mehta et al. indicated that the expression of the miR-212/132 (miR-19) cluster in HSCs derived from the bone marrow of mice increased during ageing. Inappropriate expression of these clusters led to impaired hematopoiesis with ageing. In mouse bone marrow HSCs, the overexpression of miR-19 led to the cell cycle's acceleration and destruction. Inconsistently, eliminating miR-19 expression in HSCs in response to the lack of growth factor led to disruption of the cell cycle, function and survival. The target of miR-19 is the transcription factor forkhead box O3 (FOXO3), which is one of the ageing-related genes, and miR-19 maintains the balance of hematopoietic activity by regulating the expression of FOXO3 [33].

Hu et al. revealed that deletion of homozygous bicistron miR-29a/b-1 in mouse HSPCs led to decreased self-renewal potency, with acceleration in entering the cells in cell cycle, and apoptosis of HSCs. This phenotype was reported to be caused exclusively by the loss of miR-29a, because the expression of miR-29b remained unchanged in miR-29a/b-1-null HSCs, meanwhile the ectopic expression of miR-29a alone restored HSPC function in vitro and in vivo. One of the most essential miR-29 target genes described in this study was DNA methyltransferase 3 alpha (DNMT3A), which was significantly overexpressed in miR-29a/b-1-null HSCs. This study showed that miR-29a played a critical role in maintaining HSC function through negative regulation of DNMT3A [34]. However, this is a double-edged sword because ectopic miR-29a expression in mouse HSPCs can cause a myeloproliferative disorder that progresses to acute myeloid leukemia (AML) by constructing self-renewal capacity in myeloid progenitors. Therefore miR-29a expression is reduced in hematopoietic progenitors (as opposed to HSCs) [35].

Herrera-Merchan and colleagues revealed that in super-p53 mice (carrying an extra gene dose of p53), miR-33 expression decreased HSC count with subsequent increase in MPPs. Tumor suppressor p53 regulates the cell cycle and plays an essential role in HSC self-renewal. This study showed that miR-33 led to dysregulation in the control of self-renewal by suppressing the function of p53 in mouse HSCs [36].

On the other hand, miR-125b, as an anti-apoptotic agent, leads to the expansion of early HSCs. Indeed, miR-125b overexpression is related to several hematopoietic malignancies [37]. A previous study showed that the overexpression of miR-125b led to a significant proliferation of primary B progenitor cells in the spleen and caused the expansion of the lymphoid-biased HSC subsets with anti-apoptotic activity by suppressing the expression of proapoptotic Bcl2 modifying factor (BMF) and KLF13 mRNAs. Therefore, miR-125b is related to the development of lymphoproliferative neoplasm [38].

## miRNA regulation in erythropoiesis (Table 2 and Fig. 2)

**Table 2:** miRNAs expressed in the erythroid lineage with details

Erythroid lineage	miRNA	Expression during erythroid maturation	Validated targets	Functions	References
Phase I	miR-155	Increase	MEIS-1& ETS-1↓	Inhibiting erythroid differentiation	Romania et al. [49]
Phase I	miR-152	Increase	GATA-1 ↓	Reducing hematopoiesis	Chan et al. [61]
Phase I	miR-9	Increase	FOXO3 ↓	Blocking erythroid differentiation	Zhang et al. [48]
CMP → MEP	miR-222	Increase	KIT ↓	Inhibiting normal erythropoiesis	Felli et al. [50]
MEP → BFU-e	miR-150	Decrease	Myb ↓	Blocking the commitment of MEP to the erythroid lineage	Zhou et al. [42]
MEP → BFU-e	miR-486-3p	Increase	c-MAF bZIP ↑	Increasing erythropoiesis and blocking megakaryocytopoiesis	Bianchi et al. [44]
BFU-e → CFU-e	miR-15a	Increase	Myb ↓	Inhibiting erythropoiesis	Zhao et al. [64]
BFU-e → CFU-e	miR-221	Increase	KIT ↓	Inhibiting normal erythropoiesis	Felli et al. [50]
BFU-e → CFU-e	miR-222	Increase	KIT ↓	Inhibiting normal erythropoiesis	Felli et al. [50]
CFU-e	miR-23a	Increase	GATA-1 and GATA-2	Enhancing erythroid differentiation	Zhu et al. [73]
CFU-e	miR-144	Increase	KLFD ↑, GATA-1 ↑, c-Myc ↓, RAB14 ↓	Inhibiting embryonic α-globin production (KLFD)	Dore et al. [56]; Xu et al. [57]; Fu et al. [58]; Huang et al. [83]; Xu et al. [55]; Kim et al. [59]
				Enhancing erythroid differentiation (GATA-1 & c-Myc)	
				Inhibiting embryonic β-globin production & Inhibiting maturation erythroid progenitors (RAB14)	
CFU-e	miR-451	Increase	GATA-2 ↑, c-Myc ↓, Ywhaz/14–3–3 zeta ↓, FOXO3 ↑	Enhancing erythroid differentiation (GATA-2 & c-Myc)	Dore et al. [56]; Xu et al. [57]; Huang et al. [83]; Patrick et al. [85]; Yu et al. [84]; Xu et al. [55]; Kim et al. [59]
				Protective against stress oxidative and facilitate erythroid maturation (Ywhaz/14–3–3 zeta & FOXO3)	
CFU-e → Erythroblast	miR-24	Increase	ALK4 ↓	Inhibiting normal erythropoiesis	Wang et al. [71]
CFU-e → Erythroblast	miR-16–1	Increase	Myb ↓	Leading to impaired colony formation due to decreased MYB protein levels	Sankaran et al. [65]
CFU-e → Erythroblast	miR-27a	Increase	CDC25B ↓	Promoting hemin-induced erythroid differentiation of K562 cells	Wang et al. [74]
CFU-e → Pro-erythroblast	miR-223	Increase	LMO2 ↓	Inhibiting erythropoiesis	Yuan et al. [62]; Felli et al. [50]
Erythroblast → Erythrocyte	miR-191	Inhibition	RIOK3 and Mxi1 ↓	Blocking erythroid progenitors and having minor effects on terminal erythroid proliferation or differentiation	Zhang et al. [76]
Pro-erythroblast → erythrocyte	miR-669m	Increase	Akap7 and Xk genes ↓	miR-669m inhibits late erythroblast differentiation with overexpression	Kotaki et al. [88]
Nucleation	miR-181a	Decrease	Xpo7 ↑	Leading to the occurrence of enucleation (erythroid differentiation)	Figueroa et al. [77]
Reticulocyte	miR-15b	Increase	SALL4 ↓	Leading to differentiation erythroid lineage	Choong et al. [79]; Lu et al. [80]; Aguila et al. [81]; Rahnama et al. [82]
Reticulocyte	miR-142	Increase	Rac1 ↓	Maintaining the typical erythrocyte biconcave shape and structural resilience	Rivkin et al. [89]
Reticulocyte	miR-320a	Increase	SMAR1 ↓	Inhibiting erythroid differentiation	Mittal et al. [90]
EP → RBC	miR-144	Increase	CAP1	Enucleation disruption	
Final differentiation arrest	Dore et al. [56]; Xu et al. [57]; Fu et al. [58]; Huang et al. [83]; Xu et al. [55]; Kim et al. [59]				
EP → RBC	miR-451	Increase	CAP1	Enucleation disruption	Dore et al. [52]; Xu et al. [53]; Huang et al. [79]; Patrick et al. [81]; Yu et al. [80]; Xu et al. [51]; Kim et al. [55]
				Final differentiation arrest	
Basophilic erythroblast → Polychromatic erythroblast	miR-16–2	Increase	No details	Polychromatic erythroblast differentiation	Zhang et al. [4]
No details	miR-124	Increase	Myb, TAL1 ↓	Reducing erythropoiesis	Wang et al. [67]
No details	miR-196a	Increase	p27kip1	Indicating that miR-196a overexpression inhibits apoptosis in hemin-induced K562 cells	Zhao et al. [12]
No details	miR-200a	Increase	PDCD4, THRB↓	Inhibiting erythroid differentiation	Li et al. [91]
No details	miR-218	Increase	AlAS2 ↓	Inhibiting erythroid differentiation and altering iron metabolism	Li et al. [94]
No details	miR-210	Increase	BCL11A-XL ↓	Increase γ-globin gene expression	Bianchi et al. [68]
No details	miR-376a	Decrease	CDK-2, AGO ↑	Differentiating toward the committed erythroid	Wang et al. [69]
No details	miR-17–92	Increase	TAL1↓	Inhibiting erythroid differentiation	Meyer et al. [92]
No details	miR-28	Decrease		Leading to erythroid differentiation	Kim et al. [9]
No details	miR-185	Increase		Leading to erythroid differentiation	Kim et al. [9]
No details	miR-126	Decrease	Myb ↓	Inhibiting erythroid differentiation	Huang et al. [46]; Grabher et al. [45]
No details	miR-433	Increase	GBP2 ↓	Inhibiting hematopoietic cell proliferation and erythropoiesis	Lin et al. [95]
No details	miR-Let-7d	Increase	DMT1-IRE ↓	Interference in erythroid lineage differentiation by iron accumulation in endosomes	Andolfo et al. [93]

CFU-e: Colony-forming unit-erythroid; MEP: Megakaryocyte-erythroid progenitor; EP: Erythroid progenitor; RBC: Red blood cell

**Fig. 2:**
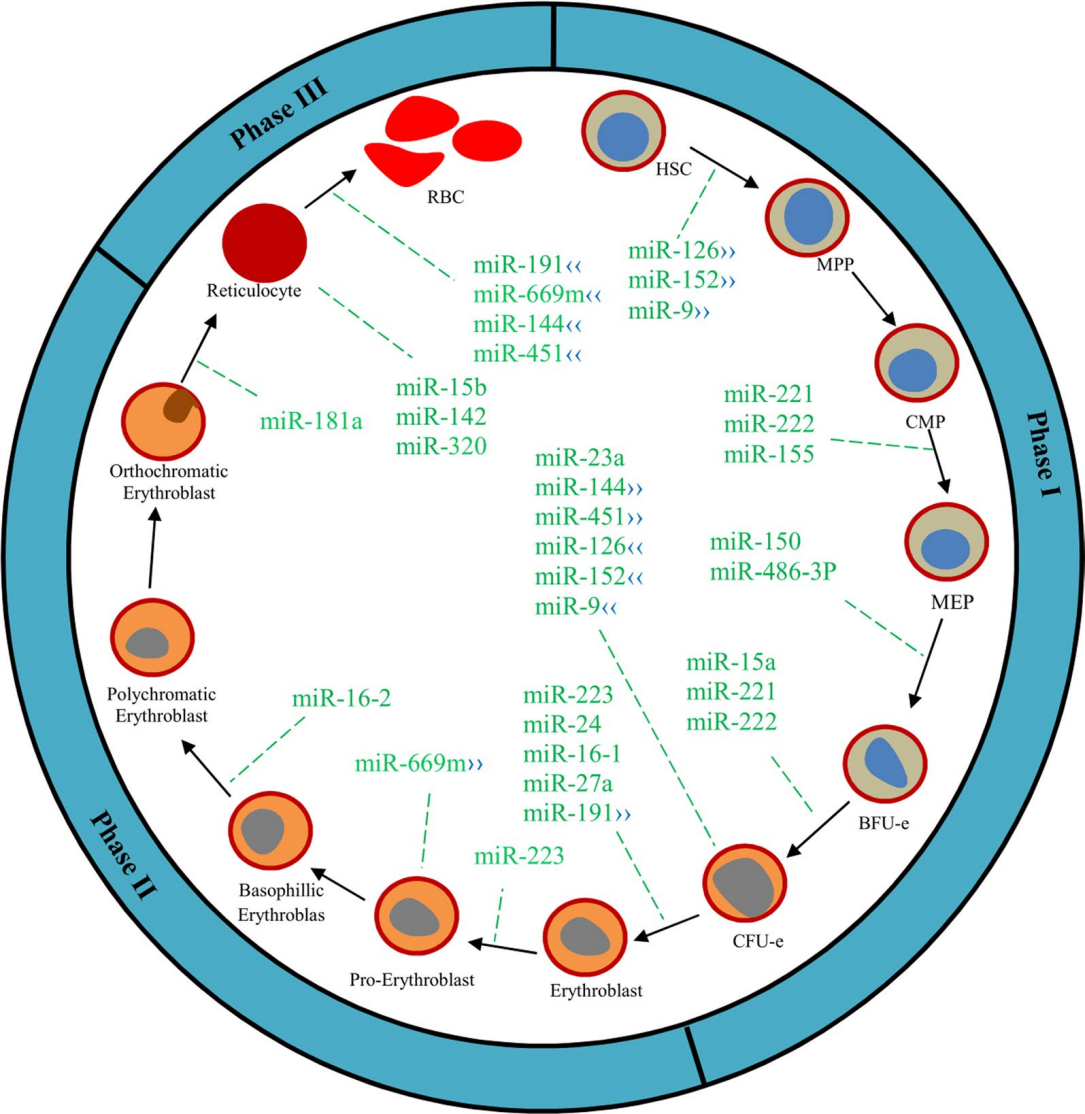
Schematic of erythropoiesis phases and some of the involved miRNAs. *Note*. HSC: Hematopoietic stem cell; MPP: Multipotent progenitor; CMP: Common myeloid progenitor; MEP: Megakaryocyte-erythroid progenitor; BFU: Blast forming unit; CFU: Colony-forming unit; RBC: Red blood cell. (›› indicates the beginning site of action & ‹‹ indicates the ending site of action)

The red blood cell (RBC) production process is well known as erythropoiesis, which includes several successive stages resulting in turning HSCs into mature RBCs. Briefly, these steps have been divided into three main phases [39]. I) HSCs → colony-forming unit-erythroid (CFU-e). First, HSCs convert to common myeloid progenitors (CMPs) and megakaryocyte-erythrocyte progenitors (MEPs). Then, they turn into burst-forming unit erythroid (BFU-E) and CFU-e. In the second phase, pro-erythroblast → orthochromatic erythroblast. In this phase, pro-erythroblasts are sequentially differentiated into basophilic, polychromatic, and orthochromatic erythroblasts. Finally, reticulocytes differentiate into mature erythrocytes (Fig. 2). Blood cells have a vital role in the body, and miRNAs are involved in each stage of their differentiation. Thus, the aberrant expression of miRNA can lead to the induction of numerous diseases. The main contribution of miRNAs during erythropoiesis is to prevent/stimulate the differentiation process [39]. Various miRNAs involved in erythropoiesis are thoroughly presented in Table 2 and then discussed based on their role in the stages of erythropoiesis.

### The first phase of erythroid differentiation (HSCs → CFU-e)

miRNAs are expressed in hematopoietic cells and a play critical role in early hematopoietic differentiation [40]. During the first phase of erythropoiesis (Fig. 2), the differentiated MEP from CMP generates BFU-e and burst-forming unit-megakaryocyte (BFU-MK), namely, erythropoiesis and megakaryocytopoiesis, respectively [41]. During this conversion, miR-150 plays a unique role in determining the fate of these two pathways through targeting Myb proto-oncogene [42] (Table 2). Myb is a proto-oncogene encoder, which is essential for lineage commitment, proliferation, and differentiation [43]. The diminished level of miR-150, as well as increased Myb, leads to the differentiation of BFU-e, in which BFU-e subsequently differentiates into CFU-e [4]. KLF17 and LIM domain only 2 (LMO2) are transcription factors that are activated under the influence of Myb and induce differentiation of erythroid lineage [44]. The Myb transcription factor results in miR-486-3p overexpression by activating the ankyrin 1 (ANK1) gene, inducing erythropoiesis, by targeting c-MAF in association with the megakaryopoiesis block [5, 44]. The knockdown of miR-126 in zebrafish hematopoietic cells increases the Myb protein level and promotes erythropoiesis; however, overexpressed miR-126 in human embryonic CD34 cells diminishes erythroid colonies [45, 46]. Regarding inconsistent reports, the existence of the MEP stage has sparked controversy [47]. HOXA9 is a transcriptional regulator that participates in regulating hematopoiesis in mice, which is in part mediated by miR-126, resulting in regulation of HOXA9 protein levels in normal hematopoiesis and prevention of leukemic transformation [9]. The erythroid differentiation has been blocked in mice erythroid precursor cells by BTG1 and CITED2 due to the miR-9 suppressing effect on the FOXO3 erythroid transcription regulator [48].

Additionally, it was reported that miR-155 could regulate erythropoiesis and myelopoiesis [7]. Overexpressed miR-155 in K562 cells reduced the differentiation of erythroid and megakaryocyte (MK) cells by inhibiting Myeloid ecotropic viral integration site 1 (MEIS-1) and Erythroblast transformation specific 1 (ETS-1) transcription factors [1, 49]. Felli et al. (2005) revealed that miR-221 and miR-222 are the first miRNAs involved in human erythropoiesis [50]. These two miRNAs were found to increase the KIT protein, resulting in reduced CD34^+^ precursor proliferation and increased erythropoietic cell differentiation [51]. BLVRA and CRKL were identified as target proteins for miR-222 by proteomics/bioinformatics analyses. The enhancement of these two proteins by inhibiting miR-222 increased erythroid differentiation from K562 cells [52]. miR-22 is another miRNA involved in MKs and erythroid cell population balance, and its expression is suppressed by SON DNA binding protein to maintain the high level of GATA-2 expression in mice [9, 53].

Myc is a proto-oncogenic protein regulating the orchestrate maturation of erythroblasts by an unknown mechanism, and an essential protein for erythroblast proliferation. It is one of the factors required to form CFU-E, differentiating into orthochromatic erythroblast [54]. miR-144 and miR-451 directly inhibit Myc in erythroblasts. Therefore, high Myc levels maintained in miR-144/451-depleted erythroblasts inhibit erythroid differentiation in mice model [55]. On the other hand, GATA-1 is one of the essential genes that regulates hematopoiesis and activates miR-144/451 [56]. Thus, the GATA-1-miR-144/451-Myc network protects the natural differentiation of the erythroid series [55]. In addition, miR-451 induction by GATA-1 induces GATA-2 overexpression, resulting in erythropoiesis progression. Xu et al. (2019) identified more than 50 new mRNAs as possible targets for miR-144/451 during mice erythropoiesis [57]. Meanwhile, Fu et al. 2010 showed that during zebrafish embryonic development, miR144 expression regulated embryonic alpha globulin gene expression by targeting the erythroid-specific KLFD. This selective pathway of globin gene regulation could be used as a new therapeutic target to improve the treatment of thalassemia [58]. Additionally, during normal human erythropoiesis, microRNAs 144 and 451 increased the expression of beta-globin and the number of erythroid cells by downregulating RAB14 [59]. In the early stages of erythroid maturation, high levels of GATA-1 transcription factor expression are essential for physiological development [60]. Hence, GATA-1 is known as a critical target for miRNAs. In *chionodraco hamatus*, miR-152 has been reported (Chan et al., 2018) to regulate GATA-1 expression, leading to hematopoiesis inhibition (Table 2) [61].

### The second phase of erythroid differentiation (pro-erythroblasts → orthochromatic erythroblasts)

Similar to the first phase, different miRNAs are involved in the second phase of erythroid differentiation (Fig. 2). The transcription factor LMO2 acts as a stimulating protein at this stage, which is inversely correlated with miR-223 [9]. LMO2 is a direct target of miR-223, in which miR-223 overexpression reduces the LMO2 protein, leading to erythroid differentiation disorder [50, 62], which causes a decrement in orthochromatophilic erythroblasts, while an increment in erythroid immature cells [63]. Undi et al. (2013) reported various proteins such as LIN54, FOXO1, USP42, ALCAM, BCLAF1, and SLC11A2 as miR-223 potential targets. However, only LMO2 was identified as a definitive target, and the others should be confirmed by further studies [1].

The importance of Myb and its association with miR-15a has been discussed in previous studies [64]. There is an active self-regulatory feedback loop between miR-15a and Myb, and their expression patterns are inversely related to each other [64]. It has been revealed that the inverse correlation between miR-15a and Myb expression levels leads to a decrement in human CD34^+^ cell differentiation when there is an increase in miR-15a expression. Along with miR-15a, miR-16–1 overexpression also causes CFU-e to pro-erythroblast differentiation arrest [63, 65]. Merkerova et al. (2008) found that miR-16 was highly expressed in most human hematopoietic cells [6]. There are similar reports regarding miR-16-5p [66]. Although miR-16–2 is associated with polychromatic erythroblast differentiation, details of their role have not been established yet [4]. In addition, miR-124 modulates the Myb protein level by targeting Myb and T-cell acute leukemia protein 1 (TAL1), resulting in erythropoiesis arrest [67]. Furthermore, miR-210 plays a key role in erythroid cell differentiation by increasing gamma-globin gene expression. It was shown that miR-210 expression during erythroid differentiation resulted in downregulation of BCL11A-XL, thereby leading to induction of gamma-globin gene expression [68]. miR-103 overexpression reduced the erythroid differentiation of K562 and CD34^+^ cells by targeting mRNA of FOXJ2 gene [69, 70].

Another involved miRNA in the intermediate stage of erythropoiesis differentiation is miR-24, which inhibits differentiation by targeting ALK4 [71]. miR-24, miR-23, and miR-27 are meaningful clusters in erythropoiesis. These clusters are also involved in the differentiation and function of other blood cells [72]. miR-23a overexpression stimulates the erythropoiesis process and increases the CFU-e capacity associated with GATA-1 [73]. Wang et al. (2018) reported that miR-27 induced K562 cell differentiation by targeting CDC25B. In other studies, it was demonstrated that GATA-1 and GATA-2 switches promoted erythropoiesis by regulating miR-24 and miR-27a expressions in zebrafish and mice HPCs [74, 75]. Considering all the aforementioned discussions, miR-24 has a contradictory role in erythroid cell differentiation [8]. Given the importance of the above-mentioned miRNA cluster, these clusters may also play a key role in other erythroid differentiation stages. However, more studies are required in this respect.

### The third phase of erythroid differentiation (Reticulocytes → RBCs)

The LMO2 transcription factor acts as a positive regulator in the early stages; however, it plays a negative regulatory role in the final stages [9]. Such evidence indicates the need for separate examinations of erythroid cell differentiation at different stages. Enucleation is one of the final stages of erythroid differentiation; during this stage, miR-191 expression reduces erythroid differentiation and proliferation by targeting RIOK3 and Mxi1 [76]. Similarly, miR-181a is also involved in enucleation, targeting exportin 7 (Xpo7). High levels of miR-181a inhibit Xpo7 expression, demonstrating a negative correlation. Thus, the miR-181a level decreases initiated Xpo7 overexpression, resulting in final nucleation in phase III [77]. The other involved miRNAs in this step, including miR-9, miR-34a, and miR-30a, suppress enucleation [77]. Ultimately, miR-199b-5p plays a unique role in the maturation of RBCs, and forced expression of miR-199b-5p in K562 cells leads to erythroid proliferation and maturation with a GATA-1- and NF-E2-dependent mechanism; in other words, GATA-1 and NF-E2 positively regulate the expression of miR-199b-5p during erythropoiesis. Moreover, it was shown by Li et al. (2014) that c-kit is another target of miR-199b-5p involved in this process [78].

miR-150, miR-155, miR-181a, and miR-342 have low expression in the final stages of erythroid differentiation [6]. miR-15b is also involved in erythroid lineage differentiation [79, 80]. Spalt-like transcription factor 4 (SALL4) is a transcription factor, capable of promoting erythroid differentiation [81]. Rahnama et al. (2015) concluded that miR-15b increases CD34^+^ cell levels by targeting SALL4 [82]. Similar to the first phase, miR-144/451 seems to be involved in the third phase. Merkerova et al. (2008) examined the expression of 13 miRNAs involved in hematopoietic cell types and reported that miR-451 was maximally expressed in reticulocytes [6]. During terminal erythropoiesis in mouse, miR-144/451 was found to have a regulatory role in enucleation by targeting cyclase-associated protein 1 (CAP1) through the regulation of actin dynamics. Indeed, increased miR-144/451 reduced CAP1 expression, resulting in final differentiation arrest [83]. The other important role of miR-451 is protecting erythroid cells against oxidative stress by repressing Ywhaz/14–3-3 zeta, a phospho-serine/threonine-binding protein that interferes with the nuclear accumulation of transcription factor FOXO3. FOXO3 positively regulates erythroid antioxidant genes and protects erythroid cells from peroxide-induced destruction [84]. Supporting this evidence, Patrick et al. (2010) found through miR-451 knockout mice that dysregulation of the expression of 14–3-3 zeta protein impaired erythroid maturation [85]. According to Zhai et al. 2014 [86], miR-146b is involved in the erythroid differentiation of CD34^+^ and K562 cells via targeting platelet-derived growth factor receptor α (PDGFRA). Moreover, miRNAs such as miR-362 and miR-188 have been identified with a potential role in erythropoiesis. However, their role has not been clarified yet [87]. During the terminal stage of erythroid differentiation in mouse (from the proerythroblast to enucleation stage), miR-669m blocks erythroid differentiation through its inhibitory effect on Akap7 and Xk genes [88].

Recent reports revealed that miR-142 plays a role in regulating erythropoiesis by targeting Rac1 [89]. Through the mechanism of actin filament homeostasis, the biconcave shape of red blood cells, as well as structural resistance/flexibility, and enucleation of RBC are maintained. Rivkin et al. (2017) also stated that erythropoiesis is disturbed in miR-142 knockout animals; therefore, by treating miR-142-/- mice with Rac1 inhibitor, the process of erythropoiesis in the bone marrow are enhanced, in particular from basophilic to acidophilic erythroblasts [89].

### Undetermined phase of erythroid differentiation

Choong et al. (2007) showed that changes in the expression of 21 miRNAs are associated with the expression of erythroid surface antigens as well as hemoglobin synthesis in human umbilical cord blood (UCB)-CD34 + cells. Among these, the expression of miR-15b, miR-16, miR-22 and miR-185 was found to strongly correlate with the expression of erythroid surface antigens (CD71, CD36 and CD235a) and hemoglobin synthesis. On the other hand, miR-28 showed an inverse correlation with the increase of all erythroid surface markers and hemoglobin synthesis [79].

The findings of Mittal et al. also demonstrated that miR-320a blocks erythroid differentiation by negatively regulating SMAR1, which regulates erythroid lineage differentiation through binding to miR-221/222 [90].

miR-376a originates from a common progenitor committed to the erythroid and megakaryocytic lineages. Overexpression of miR-376a inhibits erythroid differentiation by targeting cyclin-dependent kinase 2 (CDK2) and Argonaute 2 (Ago2) [69].

In a study by the Li et al., programmed cell death 4 (PDCD4) and thyroid hormone receptor beta (THRB) were found to participate in the differentiation of erythroid lineage, meanwhile, miR-200a inhibited the differentiation of erythroid lineage by targeting these two factors. The precise mechanism for PDCD4 and THRB is unknown and requires more investigation [91].

The miR-17–92 cluster expression decreases during terminal erythroid differentiation, and its overexpression by targeting TAL1 negatively affects erythroid lineage differentiation [92].

miR-Let-7d plays a crucial role in regulating iron metabolism in erythroid cells by targeting DMT1-IRE, as upregulation of miR-Let-7d inhibited DMT1-IRE expression in K562 and HEL cells. This miRNA can interfere with erythroid differentiation by accumulating iron in endosomes[93]. Also, miR-218 inhibits erythroid differentiation by downregulating delta-aminolevulinate synthase 2 (ALAS2) in K562 cells. ALAS2 is involved in erythroid differentiation and iron metabolism [94].

Meanwhile, overexpression of miR-433 regulates the proliferation and erythroid lineage differentiation in TF-1 cells through downregulating GBP2 [95].

## miRNA regulation in megakaryopoiesis (Table 3 and Fig. 3)

**Table 3:** miRNAs expressed in the megakaryocytic lineage with details

Megakaryocytic lineage	miRNA	Expression during megakaryocytic maturation	Transcription factor/Target	Functions	References
First phase	miR-144/451	Decrease	RUNX1 ↑	Being repressed by RUNX1 and promoting MK differentiation	Hitzler [102]
First phase	miR-138	Decrease	GATA-1 ↑	Being increased by GATA-1 and blocking MK differentiation	Xu et al. [103] & Undi et al. [1]
First phase	miR-27a	Increase	RUNX1 ↑	Being stimulated by RUNX1 and promoting MK differentiation	Ben-ami et al. [104]
First phase	miR-9	Decrease	RUNX1 ↓	Repressing RUNX1 and blocking MK differentiation	Raghuwanshi et al. [105]
First phase	miR-146a	Decrease/ Increase	PLZF ↑	Being downregulated by PLZF, which expresses CXCR4 and eventually regulates HSC homing, MK proliferation, differentiation, and maturation	Labbaye et al. [107]
				Overexpression of miR-146a could disrupt megakaryocytopoiesis	
				Inducing megakaryocytopoiesis in mice and human cell cultures	
First phase	miR-146b	Increase	PLZF ↓	Reduction in PDGFRA and promoting MK differentiation	Ferrer-Marín et al. [111]
First phase	miR-155	Decrease	ETS1 ↓	Downregulating both ETS1 and MEIS1 and inhibiting MK differentiation	Romania et al. [49]
			MEIs1 ↓		
First phase	miR-150	Increase	c-Myb ↓	Facilitating megakaryocytic differentiation	Lu et al. [41]
			TPO ↑		
First phase	miR-145	Decrease	Fli-1 ↓	Downregulating Fli-1 and inhibiting MK differentiation	Kumar et al. [116]
First phase	miR-34a	Increase	Myb ↓	Repressing the expression of Myb, CDK4, and CDK6 and facilitating megakaryocytic differentiation	Navarro et al. [118]
			CDK4 ↓		
			CDK6 ↓		
First phase	miR-142-3p	Increase	Actin cytoskeletal regulators ↑	Facilitating MK terminal differentiation	Chapnik et al. [119]
First phase	miR-10a	Decrease	HOXA1 ↑	Increasing HOXA1 expression and inhibiting MK differentiation	Zarif et al. [120]
First phase	miR-130a	Decrease	MAFB ↓	Inhibiting megakaryocytic differentiation	Garzon et al. [121]
First phase	miR-101	Decrease	MEIS2	Not being described	Garzon et al. [121]
			RUNX1		
			c-Myb		
			FOS		
			RARb		
			NFE2L2		
First phase	miR-126	Decrease	v-Crk ↑	Targeting the v-Crk gene and promoting AMKL	Garzon et al. [121]
First phase	miR-106	Decrease	RUNX1	Involved in developing MKL	Garzon et al. [121]
First phase	miR-20	Decrease	RUNX1	Involved in developing MKL	Garzon et al. [121]
First phase	miR-135	Decrease	RUNX1	Involved in developing MKL	Garzon et al. [121]
First phase & Second phase	miR-28	Decrease	MPL ↓	Suppressing the expression of the MPL gene and inhibiting MK differentiation	Girardot et al. [114] & Barroga et al. [115]
Second phase	miR-135a	Decrease	JAK2 ↑	Not being described	Navarro et al. [124]
Second phase	miR-221	Decrease	SOCS3 ↓	Reducing the expression of SOCS3	Navarro et al. [125]
Second phase	miR-203	Decrease	SOCS1 ↓	Reducing the expression of SOCS1	Navarro et al. [125]

RUNX1-: Runt-related transcription factor-1; HSC: Hematopoietic stem cell; MK: Megakaryocyte

**Fig. 3:**
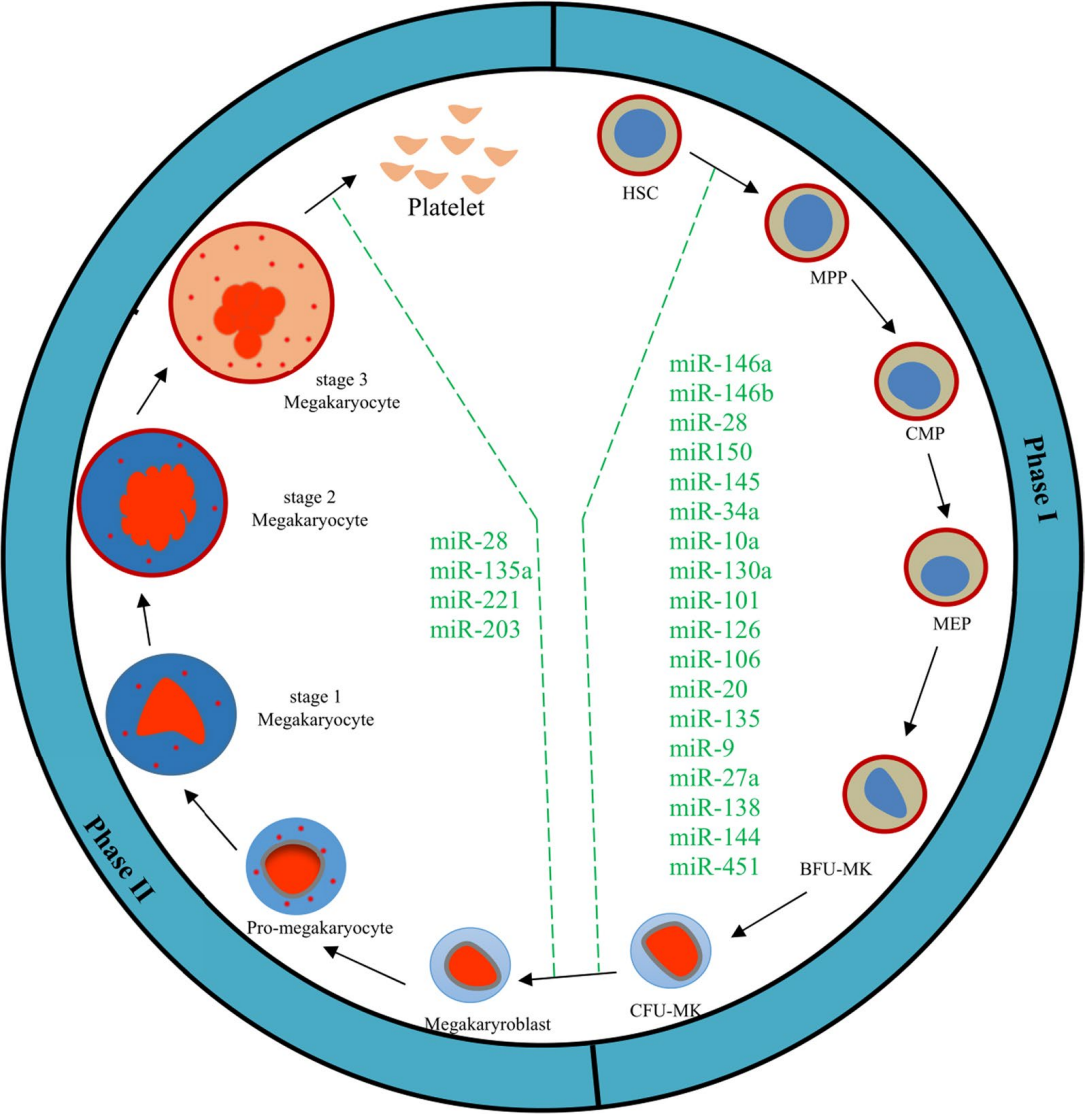
Schematic of megakaryopoiesis phases and some of the involved miRNAs. *Note*. MK: Megakaryocyte; HSC: Hematopoietic stem cell, MPP: Multipotent progenitor, CMP: Common myeloid progenitor, MEP: Megakaryocyte-erythroid progenitor; BFU: Blast forming unit; MK: Megakaryocyte, CFU: Colony-forming unit

### The first phase of MK differentiation (HSCs → CFU-Meg → MK)—Megakaryopoiesis

Megakaryopoiesis is the commitment of HSCs to the MK lineage. It can be divided into proliferative and maturation phases, in which MK precursors are multiplied and the two main events of this lineage occur, respectively. The first phase is polyploidization occurring at the nuclear level by endomitosis, and the second is cytoplasmic maturation [96].

Studies on miRNAs have shown their involvement in megakaryopoiesis by regulating transcription factors (Table 3 and Fig. 3). The miR-144/451 cluster is the most common miRNA expressed in RBCs [97]. Runt-related transcription factor-1 (RUNX1) can suppress the miR-144/451 cluster during megakaryopoiesis. It is involved in myeloid differentiation by suppressing erythroid-specific genes, simultaneously activating the transcription of MK-specific genes [98]. Moreover, RUNX1 participates in MK polyploidization [99] and cytoskeleton rearrangement in the MK maturation process [100]. On the other hand, GATA-1 activates the miR-144/451 locus [4, 56, 101], primarily stimulating erythropoiesis, especially megakaryopoiesis [102].

Accordingly, miR-138, upregulated by GATA-1, suppresses the fusion of breakpoint cluster region (BCR) and ABL1 genes. Thus, miR-138, through a BCR-ABL/GATA-1/miR-138 circuit, is a tumor suppressor miRNA which is involved in the pathogenesis of chronic myeloid leukemia (CML) and may affect the clinical response to imatinib [1, 103]. Another example of a well-established interrelation between miRNAs and transcription factors is the correlation between mirR-27a and RUNX1. miR-27a suppresses RUNX1 expression in mice. During megakaryopoiesis, miR-27a is stimulated by RUNX1. In K562, an immortal human myelogenous leukemia cell line, megakaryocytic differentiation by phorbol 12-myristate 13-acetate (PMA), results in RUNX1 to bind to a miR-27a potential regulatory region, resulting in miR-27a overexpression [104]. The interaction between RUNX1 and miRNA goes through the regulation of miRNA by RUNXI and vice versa. For instance, miR-9 can regulate RUNX1 expression. The upregulation of this miRNA in human MEG-01 and DAMI cell lines, both of megakaryoblast phenotypes, decreases RUNX1 at both mRNA and protein levels [105]. This negative correlation between RUNX1 and miR-9 has also been observed in MKs derived from the umbilical cord and peripheral blood samples [105, 106].

Promyelocytic leukaemia zinc finger protein (PLZF) is another transcription factor, which is upregulated during megakaryopoiesis. It decreases miR-146a expression, leading to C-X-C chemokine receptor type 4 (CXCR4) suppression [107]. Hence, PLZF can stimulate MK migration through the BM by promoting CXCR4 translation. ETS1 and MEIS1 are both transcription factors with well-known hematopoietic functions. ETS1 expression is increased in megakaryocytic differentiation, regulating MK-specific gene promoters such as platelet factor 4, GATA-2, or glycoprotein IIb (GPIIb) [108]. MEIS1 is an essential transcription factor for megakaryopoiesis and thrombopoiesis [109]. MiR-155 decreases ETS1 and MEIS1 expressions in human cord blood CD34 + hematopoietic progenitor cells. However, during thrombopoietin-induced megakaryocytic physiological differentiation, miR-155 expression rapidly decreases [49], facilitating the megakaryocytic differentiation process through ETS1 and MEIS1. All these studies demonstrate megakaryopoiesis regulation by miRNAs and interactions between transcription factors and miRNAs, influencing miRNA-encoding gene expression [110].

Despite transcription factors and miRNAs interactions in megakaryopoiesis, other miRNAs have also been described as complex megakaryopoiesis agents. The miR-146 family, including miR-146a and miR-146b, is suggested to be involved in hematopoiesis. In particular, miR-146a, involved in inflammatory conditions, appears to play a key role in normal hematopoiesis. Consistent with this issue, it has been revealed that deficient miR-146a mice have the phenotypic characteristics of abnormal hematopoiesis, especially BM myelofibrosis [111]. It has been elucidated that miR-146a expression upregulates during induced megakaryocytopoiesis in mice and human cell cultures. However, miR-146a expression induction has insignificant effects on the process [110]. On the other hand, it has been reported that miR-146a expression decreases when human umbilical cord CD34 + cells are enforced to differentiate into MKs [107], whereas the overexpression of miR-146a disrupts megakaryocytopoiesis [110]. In addition, miR-146a degradation in mice HSCs results in BM MK increment [112, 113]. Such a discrepancy might be due to variations in experimental conditions and diversity between the human and used mouse models. However, this issue does not dismiss the fact that miR-146a can affect megakaryopoiesis. Another member of the miR-146 family is miR-146b, which directly/indirectly contributes to the expression of PDGFRA in phorbol 12-myristate 13-acetate-differentiated K562 cells by GATA-1. miR-146b expression is increased in CD34 + hematopoietic stem/progenitor cells differentiated into MKs, associated with a simultaneous reduction in PDGFRA expression [111]. Some other studies indicated that in the differentiation processes of MKs derived from CD34 + cells, miR-28 had an adverse impact on megakaryocytic precursor differentiation. Experiments on transferring miR-28 to human CD34 + cells, using thrombopoietin (TPO), demonstrated more than 50% reductions in the number of MKs and platelets. This issue is partially defined by suppressing the expression of the MPL gene, which encodes the TPO receptor [114]. miR-150 has also been suggested to increase the TPO hormone [115]. Moreover, in vitro and in vivo experiments represented that miR-150 overexpression was associated with augmented megakaryocytic differentiation [41].

On the other hand, the increased expression of miR-145 diminishes the relative production of MKs, while the reduced expression of miR-145 increases megakaryopoiesis [116]. Kumar et al. found that miR-145 targeted the friend leukemia integration 1 (Fli-1) gene, a transcription factor playing an important role in megakaryopoiesis. They also concluded that the overexpression of Fli-1 led to myeloid malignancies. Likewise, Liu et al. (2019) reported that the miR-145 promoter was negatively regulated by Fli-1 (negative feedback) [117].

Lu et al. (2008) revealed that miR-150 regulates the differentiation of MK-erythroid progenitor cells. In experiments regarding gain and loss of function, they showed that miR-150 regulates the differentiation of MEP cells into MKs in both in vitro and in vivo conditions by modulating c-Myb [41].

The induction of miR-34a expression in K562 cells leads to cell cycle arrest in the G1 stage, subsequently inhibiting MK cell proliferation and differentiation. MiR-34a expression is also increased during the differentiation process by thrombopoietin induction in hematopoietic CD34 + precursors. Promoted CD34 + expression in these cells significantly increases the number of MK colonies. miR-34a was observed to directly regulate Myb, CDK4, and CDK6 expressions, inhibiting G1/S transmission and facilitating MK differentiation. However, miR-34a-targeted gene expression rapidly decreased after the induction of MK differentiation and before the induction of miR-34a, indicating that miR-34a was not involved in their initial regulation. Conversely, it has been suggested that miR-34a probably helped maintain the suppression of these target genes at later stages [118].

Chapnik et al. (2014) showed that miR-142 was highly expressed in the adult hematopoietic system, highlighting their key role in megakaryopoiesis. A genetic defect in miR-142 led to impaired MK maturation, inhibited polyploidization, abnormal platelet formation, and thrombocytopenia. Their findings confirmed the prominent role of miR-142 activity in the maturation and function of MKs. A lack of miR-142 resulted in impaired myeloerythroid lineage, incomplete MK maturation, and impaired actin cytoskeletal dynamics. During megakaryopoiesis, miR-142-3p targets some actin cytoskeletal regulators to facilitate pro-platelet formation [119].

Zarif et al. (2013) reported that miR-10a could adversely affect HOXA1 mRNA expression. The HOXA1, as an inhibiting transcription factor, blocks the MK differentiation of stem cells. However, the downregulation of miR-10a increased the MK differentiation of stem cells by downregulating HOXA1 [120].

miR-130a expression decreases during megakaryocytic differentiation. The functional analysis of miR-130a demonstrated that this miRNA could suppress the MAFB by upregulating GPIIb mRNA expression, synergically with GATA-1, SP1, and ETS-1 [121].

According to the above-mentioned study, miR-101, miR-126, miR-106, miR-20, and miR-135 expression decrease during megakaryocytic differentiation. However, their expressions increase in the AMKL cell lines. They suggested that miR-101, miR-106, miR-135, and miR-20 might be involved in developing MKL by targeting the RUNX1 gene, the most frequent gene involved in leukemia. Based on their results, miR-126 could play a role in MKL by targeting the v-Crk gene, which is an oncogene product [121].

### The second phase of MK differentiation (MK → Platelet)—Thrombopoiesis

During the thrombopoiesis process, MKs generate platelets. In this process, microtubules elongate MK extensions and transfer the granules from MKs to developing platelets [122]. Many of the above-mentioned transcription factors are also involved in thrombopoiesis, making miRNAs regulating these transcription factors also affect thrombopoiesis considering that many of described mutations in RUNX1, Fli-1, GATA-1, GFI1B, ETV6, EVI1, and HOXA11 are associated with variable thrombocytopenia [123]. These transcription factors which encode gene suppression/mutations cause a functional loss in their proteins. Furthermore, miRNAs can partially regulate megakaryopoiesis and platelet formation.

Moreover, miRNAs regulating the expression of genes that encode Janus kinase (JAK), as well as the signal transducer and activator of the transcription (STAT) pathway, a key pathway for controlling platelet homeostasis, are discussed in this section. In this respect, it has been revealed that mutations in the genes of this pathway, including JAK2V617F in JAK2 and MPL exon 10, lead to a myeloproliferative neoplasm labeled essential thrombocythemia (ET) [96]. Accordingly, miR-28 has been reported to target the 3' UTR MPL region and repress its translation, potentially decreasing the number of MKs and platelets [114]. The JAK2 regulation by miRNAs in the thrombopoiesis is unidentified; however, Navarro et al. found the direct regulation of JAK2 by miR-135a in Hodgkin’s lymphoma by targeting the 3' UTR region [124]. Navarro et al. (2016) also confirmed the regulatory impact of miR-203 and miR-221 on suppressor of cytokine signaling 1 (SOCS1) and suppressor of cytokine signaling 3 (SOCS3) (negative regulators of the JAK/STAT pathway), respectively. Additionally, the expression levels of these two miRNAs were inversely correlated with SOCS1 and SOCS3 levels in the platelets of patients with ET. These findings indicate that in combination with epigenetic regulation, these miRNAs could reduce the expression of SOCS1 and SOCS3 in JAK2V617-negative patients with ET when the JAK/STAT pathway is activated [125].

In addition to the JAK/STAT pathway, Rowley et al. (2016) showed that the MK of the murine model with the specific knockdown of Dicer1, the ribonuclease that cleaves miRNA precursors into mature miRNAs, decreased the level of mostly identified miRNAs in platelets, which was associated with mild thrombocytopenia [126].

## miRNA regulation in granulocytopoiesis (Table 4 and Fig. 4)

**Table 4:** miRNAs expressed in the granulocytic lineage with details

miRNA	Expression during granulocytic differentiation	Transcription factor/target	Functions	References
miR-223	Increase	NFI-A ↓	NFI-A decreases miR-223 expression	Fazi et al. [128], Nervi et al. [129], Garzon et al. [130], Fukao et al. [131] & Johnnidis et al. [132]
		C/EBPalpha ↑	C/EBPalpha increases miR-223 expression	
		Mef2C ↓	Mef2C, which promotes myeloid proliferation, is targeted by miR-223	
miR-21	Decrease	Gfi1 ↑	Regulation of miR-21 expression	Velu et al. [133]
miR-196B	Decrease	Gfi1 ↑	Regulation of miR-196B expression	Velu et al. [133]
miR-27	Increase	AML1 ↓	AML1 targeting and a decrease in its expression	Feng et al. [134]
miR-382-5p	Increase	MXD1 ↑	Targeting MXD1 leading to differentiation of CD34 + HSCs into the granulocyte lineage	Zini et al. [135]

**Fig. 4:**
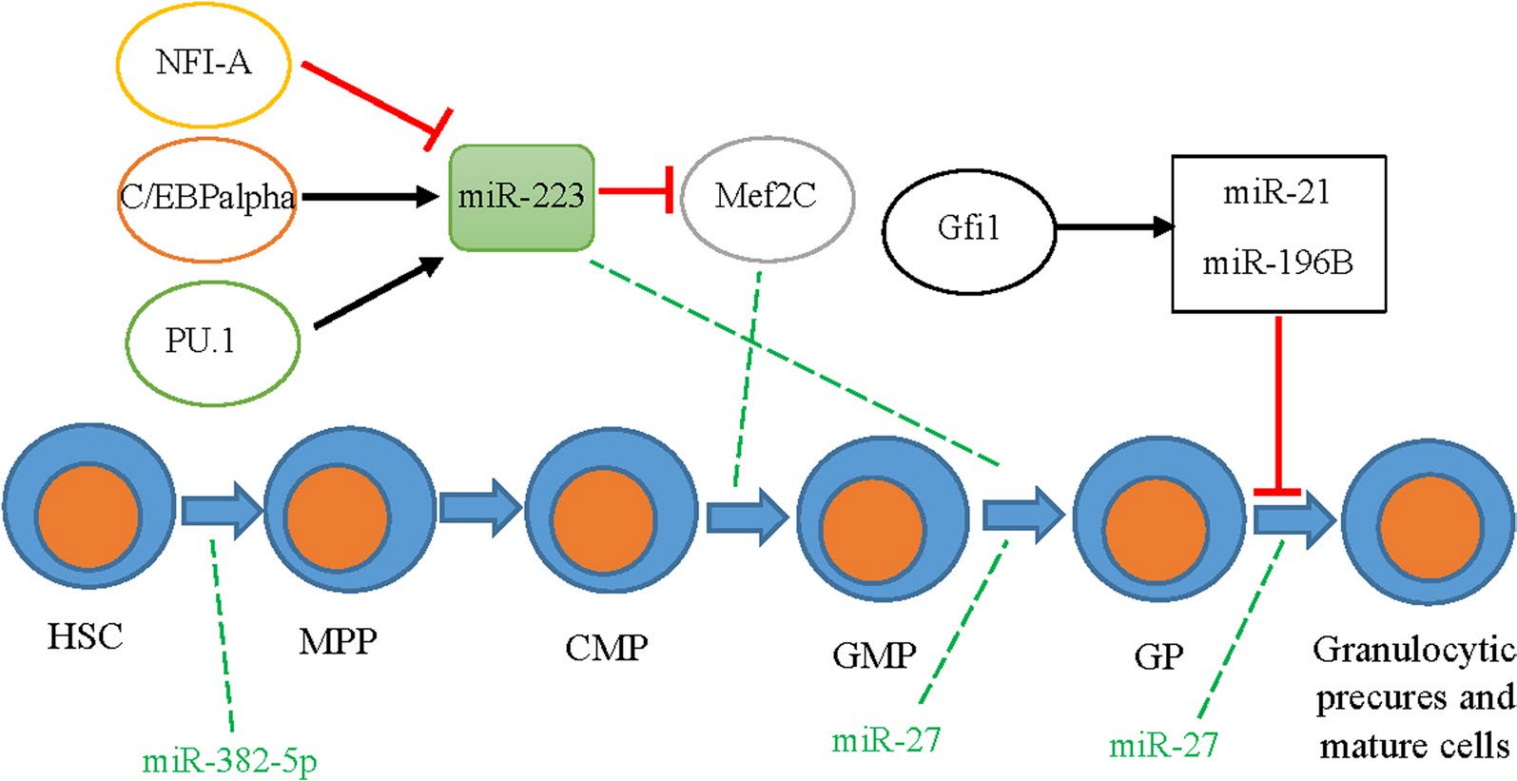
Schematic of granulocytopoiesis and some of the involved miRNAs. *Note*. HSC: Hematopoietic stem cell; MPP: Multipotent progenitor; CMP: Common myeloid progenitor; GMP: Granulocyte-monocyte progenitor; GP: Granulocyte progenitor

The first step during granulo-monocytic commitment is CFU-GEMM. In the next stage of differentiation, some progenitor cells are formed, including the granulo-monocytic CFU, the monocyte/macrophage/dendritic colony-forming unit, and the granulocyte colony-forming unit. According to previous works [127], transcription factors involved in myeloid cell production are PU.1, CCAAT/enhancer-binding proteins (C/EBP), interferon regulatory factor-8, RUNX1 (also known as AML1), and stem cell leukemia factor (also known as TAL1).

miR-223 is one of the essential miRNAs involved in granulocytic differentiation, where its expression is low in hematopoietic progenitor cells and remarkably increases during granulocytic differentiation [128]. A significant increase in miR-223 expression has been observed in acute promyelocytic leukemia (APL) patients with a retinoic acid prescription. This increment led to granulocytic differentiation induction [128–130]. In a study on the miR-223 gene promoter, a regulatory sequence was discovered, to which nuclear factor 1 A-type (NFI-A) and C/EBPalpha, two well-known transcription factors, would bind competitively [128]. According to these researchers, the binding of NFI-A keeps miR-223 expression at a low basal level; however, C/EBPalpha significant binding increases miR-223 expression. It has been explained that APL treatment with retinoic acid resulted in the replacement of NFI-A with C/EBPalpha, leading to increased miR-223 expression, which, in turn, suppressed NFI-A translation. Finally, APL cells increased their granulocytic differentiation capacity by increasing miR-223 expression. This evidence suggests that miR-223 is a positive modulator of granulocytic differentiation (Fig. 4 and Table 4) [128–130].

Other studies reported further details on the role of miR-223 in granulopoiesis. In one study, Fukao et al. (2007) found that PU.1 regulated miR-223 expression during granulocytic differentiation through retinoic acid induction [131]. Johnnidis et al. (2008) also reported a developed granulocytic compartment in mice models with miR-223 mutation as a result of the spontaneous proliferation of progenitor cells. It has been also suggested that miR-223 directly targeted Mef2C, a myeloid proliferation-promoting transcription factor capable. miR-223 ablation in granulocytes resulted in hypermature cells with increased fungicidal activity (Fig. 4) [132].

Another study introduced zinc finger protein growth factor independent-1 (Gfi1) as one of the particular regulators of miRNAs in the granulocytic lineage. The BM cells of Gfi1-/- mice or patients with mutant Gfi1 showed a defective regulation of miR-21 and miR-196B expression. On the other hand, the overexpression of these two miRNAs suppressed the G-CSF-induced granulocytic differentiation in these cells [133]. The other miRNA involved in granulocytic differentiation is miR-27, whose expression increases during granulocyte differentiation (Fig. 4). It has recently been revealed that the transcription factor AML1 is the target of this miRNA in granulocyte cells, in which the expression of this transcription factor sharply decreases during granulocytic differentiation [134].

Zini et al. showed that overexpression of miR-382-5p in human CD34 + HSCs led to a significant reduction of megakaryocyte progenitors and an increase in the granulocyte lineage. The key target factor for miR-382-5p was found to be MAX dimerization protein 1 (MXD1), through its regulation, this miRNA results in differentiation of CD34 + HSCs into granulocyte lineage while simultaneously blocks megakaryocytic differentiation [135].

## miRNA regulation in monocytopoiesis (Table 5 and Fig. 5)

**Table 5:** miRNAs expressed in the monocytic lineage with details

Monocyte lineage	miRNA	Expression in monocyte	Validated targets	Functions	References
HSCs → MPPs	miR-146a	Increase	PU.1↑	Monocyte/macrophage development	Ghani et al. [150]
	miR-155				Xu et al. [151]
	miR-342				
	miR-338				
HSC → MPP	miR-17–92 cluster	Decrease	Egr2 and PU.1 ↑	Macrophage differentiation	Pospisil et al. [153]
Phase III					
MPP → GMP	miR-142-3p	Increase	cyclin T2 ↓	Inhibiting monocyte differentiation	Wang et al. [157]
MPP → GMP	miR-142-3p	Increase	TAB2↓	Leading to monocyte/granulocyte differentiation	Wang et al. [157]
MPP → GMP	miR-223	Decrease	CACTIN, Ube2g2, CARM-1, and Ndufaf6 ↑	Macrophage differentiation	M'Baya-Moutoula et al. [154]
CMP → GMP	miR-22	Decrease	Gfi1↑	Macrophage activation	Velu et al. [133]
	miR-196b				
CMP → GMP	miR-29a	Increase	cyclin T2 ↓	inhibiting monocyte differentiation	Wang et al. [157]
CMP → GMP	miR-29a	Increase	CDK6↓	Leading to monocyte/granulocyte differentiation	Wang et al. [157]
GMP → MP	mir17-5p–20a–106a	Increase	AML1 ↓	Enhanced blast proliferation and inhibition of monocytic differentiation and maturation	Fontana et al. [141]
GMP → MP	miR-17-5p	Decrease	Hif-1α and -2α↑	Differentiation of primary human monocytes into macrophages	Poitz et al. [170]
	miR-20a				
GMP → Monocyte	miR-196	Increase	HOXB8 ↓	Enhanced myeloid differentiation of HL60 cells	Kawasaki & Taira [145]
GMP → Monocyte	miR-181	Increase	PRKCD, CTDSPL, ،CAMKK1↓	Inhibition of macrophage differentiation of HL-60 & CD34 +	Su et al. [156]
GMP → Monocyte	miR-424	Increase	PU.1 and NFI-A ↓	Monocyte/macrophage differentiation regulation	Rosa et al. [152]
Monocyte → Macrophage	miR-15a	Decrease	IKKalpha	Monocyte-macrophage differentiation	Li et al. [163]
Monocyte → Macrophage	miR-16	Decrease	IKKalpha	Monocyte-macrophage differentiation	Li et al. [163]
Phase III	miR-200b-3p	Increase	p38 MAPK ↑	Promotion of monocyte-macrophage differentiation	Yu et al. [168]
Phase III	miR-129	Increase	RUNX1↓	Promotion of monocyte-macrophage differentiation	Zhao et al. [146]
Phase III	miR-199a-5p	Increase	Activin A type 1B receptor gene	Inhibition of monocyte/macrophage differentiation	Lin et al. [165]
No details	miR-125b	Increase	ABTB1 and CBFB ↓	Blockage of apoptosis and myeloid differentiation	Bousquet et al. [167]
No details	miR-150-5p	Decrease	PU.1 ↑	Regulation of macrophage differentiation and function	Shakerian et al. [171]
No details	miR-22	Increase	EVI1 ↓	Regulation of monocyte/macrophage differentiation	Shen et al. [155]
No details	miR-125b	Increase	Stat3 and Bak1 ↓	Promotion of myelopoiesis	Surdziel et al. [166]

RUNX1: Runt-related transcription factor-1; GMP: Granulocyte-monocyte progenitor; MP: Monocyte progenitor

**Fig. 5:**
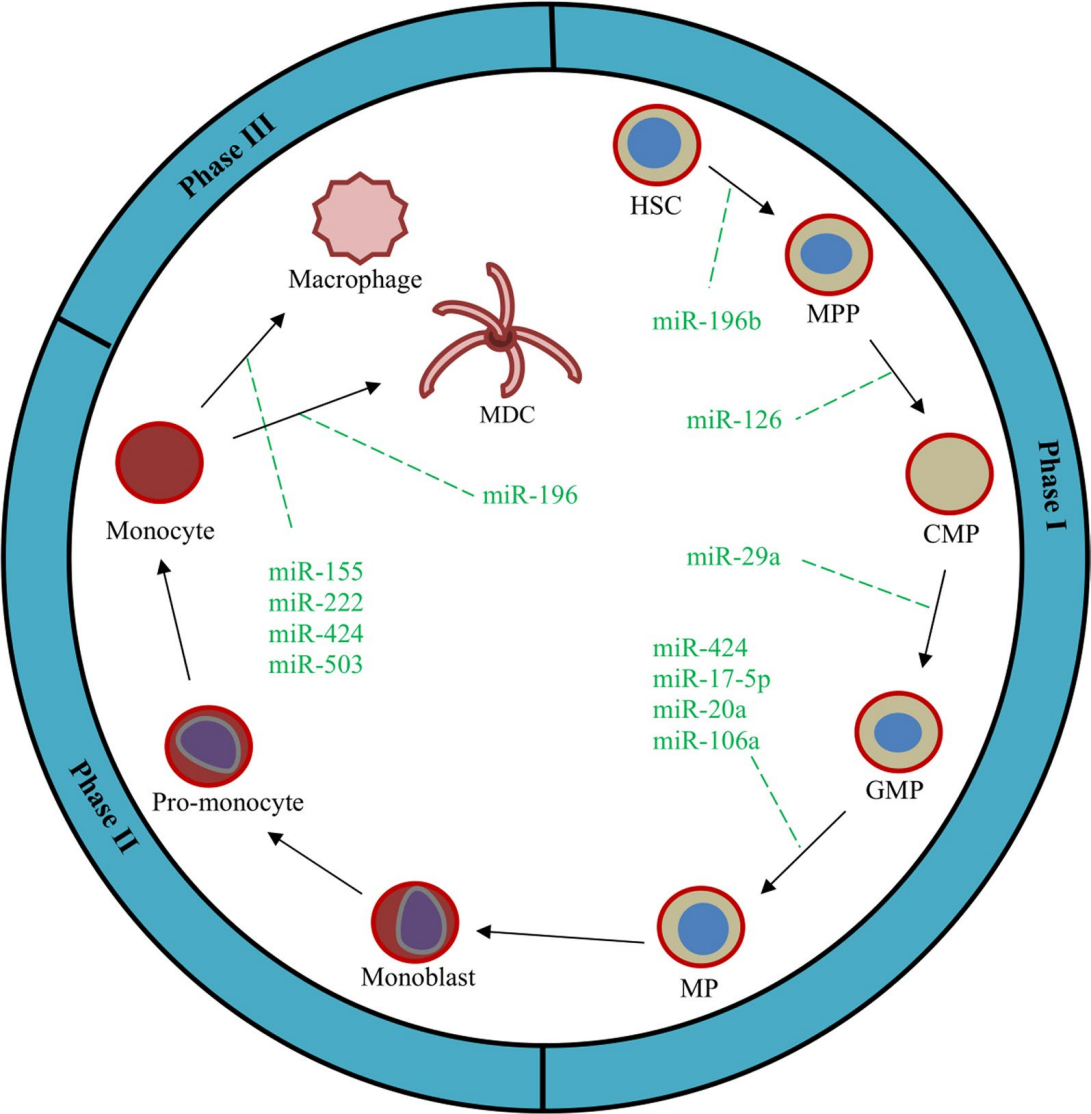
Schematic of monocytopoiesis phases and some of the involved miRNAs. *Note*. HSC: Hematopoietic stem cell; MPP: Multi-potent progenitor; CMP: Common myeloid progenitor; GMP: Granulocyte-monocyte progenitor; MP: Monocyte progenitor, MDC: Myeloid dendritic cell

Monocytopoiesis is a process in which monocytes and macrophages differentiated from progenitor cells. Although there is no clear distinction between the stages of monocytopoiesis, similar to erythropoiesis, they can be divided into three phases (Fig. 5 and Table 5). Monoblasts are the first recognizable cells in the monocytic series [136]. In the next step, the promonocytes, which are larger cells, differentiate from monoblasts [137], and finally, monocytes are produced from promonocytes. Then, monocytes can differentiate into macrophage and myeloid dendritic cells (Fig. 5). Various miRNAs are present during the stages of myelopoiesis [138–140].

Monocytopoiesis is controlled by a circuit including various miRNAs such as miR-17-5p, miR-20a, and miR-106a. These miRNAs target AML1 [141]. AML1 promotes monocytic differentiation by activating macrophage colony-stimulating factor (M-CSF), an essential growth factor for monocyte differentiation [142]. The combination of miR-20a, miR-106a, and miR-17-5p down-regulates M-CSF receptor expression. This reduction results in inhibited monocyte differentiation and maturation and enhances blast proliferation [141, 143]. Examining the pattern of miRNAs in monocytes, dendritic cells, and macrophages, Tserel et al. (2011) reported that monocytes are more likely to convert to macrophages than dendritic cells [144].

In one of the first studies conducted by Kawasaki and Taira (2004), it has been stated that miR-196 increased the myeloid differentiation of HL-60 cells by inhibiting homeobox B8 (HOXB8) expression [145]. The results of the RNA-seq technique showed that miR-129 blocks RUNX1 transcription factor expression, resulting in monocyte-macrophage differentiation [146]. Monocyte-to-macrophage differentiation is a complex biological process regulated by several transcription factors such as mTOR, Myb, STAT1, CTNNB, and E2F [147]. Meanwhile, during myeloid differentiation, the levels of transcription factors PU.1 and C/EBPalpha were involved in monocytic versus granulocytic selection. Indeed, the PU.1 transcription factor was found to be a key switch in monocyte/macrophage development, where high expression of PU.1 resulted in monocyte development. In contrast, the low expression of PU.1 led to the development of granulocytes [148]. Conversely, another study reported that PU.1 expression was increased during granulocyte and macrophage differentiation, while it diminished in T and B lymphocyte differentiation [149]. In macrophage development, PU.1 regulates the expression of at least four miRNAs, including miR-146a, miR-342, miR-338, and miR-155 [150] and plays an important role in the differentiation of HSC to MMP [151]. Rosa et al. (2007) concluded that miR-424 with PU.1 interaction by targeting NFI-A led to monocyte/macrophage differentiation. NFI-A plays an important role in the differentiation commitment of granulocyte and monocyte [152]. The miR-17–92 cluster encodes around 15 miRNAs, some of which control macrophage differentiation in interaction with PU.1 and Egr2 transcription factors. The expression of this cluster increases in primary stem cells and precursors, but it decreases with the initiation of myeloid differentiation [153].

Moutoula et al. (2018) showed that the change in the expression of miR-223 through the NF-kB-dependent pathway led to a change in the expression of CACTIN, coactivator associated arginine methyltransferase 1 (CARM-1), monocyte chemoattractant protein-1 (MCP-1) and ubiquitin conjugating enzyme E2 G2 (Ube2g2) proteins, thereby resulting in the differentiation of monocytes into macrophages as well as osteoclastogenesis. Furthermore, they revealed that RAW 264.7 cells differentiated into macrophage type I by reducing NF-kB levels, via a marginal effect on CARM-1 and CACTIN levels, while reducing MCP-1, NADH:ubiquinone oxidoreductase complex assembly factor 6 (Ndufaf6) and Ube2g2 levels. Also, by differentiating into macrophages type II, miR-223 significantly increased the levels of NF-kB with a minor effect on the levels of CACTIN, MCP-1, Ndufaf6, and Ube2g2 [154].

Moreover, Shen et al. (2016) demonstrated that miR-22 had a role in monocyte/macrophage differentiation by targeting EVI1. miR-22 inhibits EVI1, resulting in a decrease in GATA2 expression and an increase in c-Jun. Moreover, it subsequently increases the interaction between c-Jun and PU.1, decreases the interaction between MECOM and GATA2 and finally causes monocyte/macrophage differentiation [155].

Su et al. (2015) also showed that miR-181a induced granulocyte and macrophage differentiation of HL-60 and CD34 + HSPCs through targeting three genes, including PRKCD (which then affected the PRKCD-P38-C/EBPalpha pathway), CTDSPL (which then affects retinoblastoma protein phosphorylation) and CAMKK1. miR-181 inhibited macrophage differentiation by reducing the expression of all these genes. Also, this study showed that in patients with AML, myeloid differentiation is reversed by inhibiting the miR-181 family with subsequent increase in the expression level of target proteins [156].

Gfi1 has an essential role during normal granulocyte differentiation. It has been revealed that in the granulocyte and monocytic differentiation models, miR-21 and miR-196b expression is constantly in contrary with Gfi1. In other words, during the differentiation of CMP to GMP, the increase in Gfi1 expression downregulates the miR-22 and miR-196b [133].

miR-29a and -142-3p are pivotal for regulating normal myeloid differentiation. They both repress the cyclin T2 gene, prevent the release of hypophosphorylated retinoblastoma and inhibit the monocytic differentiation. Wang et al. (2012) showed that cyclin-dependent kinase 6 (CDK6) and TGF-β activated kinase 1/MAP3K7 binding protein 2 (Table 2) are under the control of miR-29a and 142-3p during monocytic and granulocytic differentiation. They revealed that enforced expression of both miRNAs decreased target protein expression and induced myeloid differentiation [157].

The expression of miR-155, miR-222, miR-424, and miR-503 is also involved in monocytic differentiation through combinatorial regulation [158, 159]. A study showed that the overexpression induction of these miRNAs by PMA led to cell cycle arrest with subsequent differentiation. Typically, their combined induction regulation increased monocyte differentiation [158]. miR-155, miR-146a, and miR-21 are associated with macrophage activation [160]. Some reports indicated that miR-223 led to monocyte-macrophage differentiation by downstreaming gene regulation [161, 162]. In addition, miR-15a and miR-16 expressions are decreased during human monocyte-macrophage differentiation, resulting in IKKalpha overexpression in macrophages. In the next process, p52 production increases and prevents a new macrophage activation by suppressing NF-κB target genes [163]. miR-15a has been found to inhibit the formation of myeloid cells [164].

During the monocyte/macrophage differentiation of HL-60, THP-1, and CD34 + cells, miR-199a-5p has a down-regulatory role by targeting the activin A type 1B receptor gene. Subsequently, the downregulation of the activin A type 1B receptor gene leads to a reduction in C/EBPalpha expression and ultimately inhibits monocyte/macrophage differentiation. miR-199a-5p can also upregulate the PU.1 transcription factor [165]. Moreover, by targeting STAT3 and BAK1, miR-125b reduces the expression of these proteins by 30% and 50%, respectively, resulting in the accumulation of myelopoiesis in mouse BM chimeras [166].

Some miRNAs can transform myeloid cell lines. For instance, Bousquet et al. (2012) reported that miR-125b targeted ABTB1 and CBFB [167]. This miRNA could also control apoptosis by down-regulating BAK1 and TP53INP1 genes. Additionally, miR-200b-3p from the miR-200 family promoted monocyte-macrophage differentiation in humans by stimulating p38 MAPK [168]. In addition to mammals, it was represented that gga-miR-200b-3p promotes macrophage activation and differentiation in birds [169]. The expression of Hif-1 and Hif-2 transcription factors significantly changes during the differentiation of monocytes to macrophages, which is controlled by miR-17 and miR-20a [170]. Shen et. al [155] also indicated that PU.1-modulated miR-22 is a monocyte/macrophage differentiation regulator. In association with macrophage differentiation and activation, miR-150 downregulates PU.1, decreases the expression of proinflammatory cytokines, and turns the polarization of macrophages away from the M1-like phenotype (inflammatory response) [171]. Future studies may shed light on the interactive role of these complex miRNA series during monocytic commitment in a time-resolved manner.

## Conclusion

miRNAs, which were discovered for the first time in a genetic study on nematodes, have become one of the main topics in human genetics in recent years. Based on findings in the last two decades, miRNAs’ role in hematopoiesis has become blatant. MiRNAs get involved in regulating hematopoietic cell growth and development by regulating transcription factors, growth factor receptors, and regulatory signals. The exact mechanism of these regulations is not clear yet. However, it has been suggested that miRNAs probably modulate hematopoiesis by the suppression or stimulation of proteins involved in critical signaling pathways. Although several studies have confirmed a significant relationship between miRNA dysregulation and leukemias and lymphomas, more studies are required to clarify the function of miRNAs in hematopoiesis and cancer. Comprehending the interaction between miRNAs and the transcriptome, including coding and non-coding RNAs, there should be significant efforts to determine how miRNAs regulate genes expression and innovation miRNA-based therapeutic techniques.

## Data Availability

Not applicable.

## Abbreviations

ABTB1: Ankyrin repeat and BTB (POZ) domain containing 1
Akap7: A-kinase anchoring protein 7
ALAS2: Delta-aminolevulinate synthase 2
ALK4: Activin-like kinase 4
ALCAM: Activated leukocyte cell adhesion molecule
AMKL: Acute megakaryoblastic leukemia
AML1: Acute myeloid leukemia 1
ANK1: Ankyrin 1
APL: Acute promyelocytic leukemia
BAK1: BCL2 Antagonist/Killer 1
BCLAF1: BCL2 associated transcription factor 1
BCL11A: B-cell lymphoma/leukemia 11A
BCR: Breakpoint cluster region
BFU: Burst-forming unit
BLVRA: Biliverdin reductase A
BM: Bone marrow
BMF: Bcl2 modifying factor
BTG1: B-cell translocation gene 1
CAP1: Cyclase-associated protein 1
CARM-1: Coactivator associated arginine methyltransferase 1
CBFB: Core-binding factor subunit beta
CDC25B: Cell division cycle 25B
CDK: Cyclin dependent kinase
C/EBP: CCAAT/enhancer-binding protein
CFU: Colony-forming unit
CFU-GEMM: Colony forming unit granulocyte, erythroid cell, monocyte, and megakaryocyte
CITED2: Cbp/P300 Interacting transactivator with Glu/Asp rich carboxy-terminal domain 2
CML: Chronic myeloid leukemia
CMP: Common myeloid progenitor
CRKL: CRK-like protein
CTNNB: Catenin (cadherin-associated protein) beta
CXCR4: C-X-C chemokine receptor type 4
DMT1: Divalent metal transporter 1
DNMT3A: DNA methyltransferase 3 alpha
EIF4G2: Eukaryotic translation initiation factor 4 gamma 2
ET: Essential thrombocythemia
ETS-1: Erythroblast transformation specific 1
ETV6: ETS variant transcription factor 6
EVI1: Ecotropic viral integration site 1
Fli-1: Friend leukemia integration 1
FOXJ2: Forkhead box protein J2
FOXO1: Forkhead box O1
FOXO3: Forkhead box O3
GBP2: Guanylate-binding protein 2
G-CSF: Granulocyte colony-stimulating factor
Gfi1: Growth factor independence-1
GFI1B: Growth Factor Independent 1B
GPIIb: Glycoprotein IIb
HMGA2: High mobility group A2
HOXB8: Homeobox B8
HOXA9/10/11: Homeobox A9/10/11
HSC: Hematopoietic stem cell
HSPC: Hematopoietic stem-progenitor cell
IEG: Immediate early genes
IRAK1: Interleukin 1 receptor associated kinase 1
IRE: Iron response element
JAK: Janus kinase
KLF: Kruppel-like factor
LMO2: LIM domain only 2
MAFB: V-maf musculoaponeurotic fibrosarcoma oncogene homolog B
MAPK: Mitogen-activated protein kinase
MCP-1: Monocyte chemoattractant protein-1
M-CSF: Macrophage colony-stimulating factor
MEIS-1: Myeloid ecotropic viral integration site 1
MEP: Megakaryocyte-erythrocyte progenitor
miRNA: MicroRNA
MK: Megakaryocyte
MKL: Megakaryoblastic leukemia
MPL: Myeloproliferative leukemia protein
MPP: Multipotential progenitor
MXD1: MAX dimerization protein 1
Mxi1: Max interactor 1
Ndufaf6: NADH:ubiquinone oxidoreductase complex assembly factor 6
NF-E2: Nuclear factor-erythroid 2
NFI-A: Nuclear factor 1 A-type
NF-κB: Nuclear factor kappa B
NR4A1: Nuclear receptor 4A1
PDCD4: Programmed cell death 4
PDGFRA: Platelet-derived growth factor receptor α
PLZF: Promyelocytic leukaemia zinc finger protein
RIOK3: RIO kinase 3
RUNX1: Runt-related transcription factor-1
SALL4: Spalt-like transcription factor 4
SLC11A2: Solute carrier family 11 member 2
SMAR1: Scaffold/matrix-associated region-binding protein 1
SOCS1: Suppressor of cytokine signaling 1
SOCS1: Suppressor of cytokine signaling 3
SP1: Specificity protein 1
STAT: Signal transducer and activator of the transcription
TAL1: T-cell acute leukemia protein 1
TET2: Tet methylcytosine dioxygenase 2
TGF-β: Transforming growth factor beta
THRB: Thyroid hormone receptor beta
TPO: Thrombopoietin
TP53INP1: Tumor protein p53 inducible nuclear protein 1
TRAF6: TNF receptor associated factor 6
Ube2g2: Ubiquitin conjugating enzyme E2 G2
USP42: Ubiquitin specific peptidase 42
UPS: Ubiquitin–proteasome system
Xk: X-linked Kx blood group
Xpo7: Exportin 7
ZFP36: Zinc finger protein 36
